# Deep learning approach to classification of lung cytological images: Two-step training using actual and synthesized images by progressive growing of generative adversarial networks

**DOI:** 10.1371/journal.pone.0229951

**Published:** 2020-03-05

**Authors:** Atsushi Teramoto, Tetsuya Tsukamoto, Ayumi Yamada, Yuka Kiriyama, Kazuyoshi Imaizumi, Kuniaki Saito, Hiroshi Fujita

**Affiliations:** 1 Faculty of Radiological Technology, School of Medical Sciences, Fujita Health University, Toyoake, Japan; 2 School of Medicine, Fujita Health University, Toyoake, Japan; 3 Department of Electrical, Electronic and Computer Engineering, Faculty of Engineering, Gifu University, Gifu, Japan; University of Oklahoma, UNITED STATES

## Abstract

Cytology is the first pathological examination performed in the diagnosis of lung cancer. In our previous study, we introduced a deep convolutional neural network (DCNN) to automatically classify cytological images as images with benign or malignant features and achieved an accuracy of 81.0%. To further improve the DCNN’s performance, it is necessary to train the network using more images. However, it is difficult to acquire cell images which contain a various cytological features with the use of many manual operations with a microscope. Therefore, in this study, we aim to improve the classification accuracy of a DCNN with the use of actual and synthesized cytological images with a generative adversarial network (GAN). Based on the proposed method, patch images were obtained from a microscopy image. Accordingly, these generated many additional similar images using a GAN. In this study, we introduce progressive growing of GANs (PGGAN), which enables the generation of high-resolution images. The use of these images allowed us to pretrain a DCNN. The DCNN was then fine-tuned using actual patch images. To confirm the effectiveness of the proposed method, we first evaluated the quality of the images which were generated by PGGAN and by a conventional deep convolutional GAN. We then evaluated the classification performance of benign and malignant cells, and confirmed that the generated images had characteristics similar to those of the actual images. Accordingly, we determined that the overall classification accuracy of lung cells was 85.3% which was improved by approximately 4.3% compared to a previously conducted study without pretraining using GAN-generated images. Based on these results, we confirmed that our proposed method will be effective for the classification of cytological images in cases at which only limited data are acquired.

## Introduction

Lung cancer is the leading cause of death among men worldwide [1]. According to the pathological examinations performed to provide detailed lung cancer diagnoses, it has become possible to identify tissue types and subtypes via immunostaining and genetic examinations [2]. Based on these tests, patients may undergo surgery, radiation therapy, drug therapy, or a combination of these treatments. With the advent of molecular targeting drugs and immune checkpoint inhibitors [3], good therapeutic results have been obtained in recent years, and accurate diagnoses have thus become essential for determining appropriate therapeutic methods.

In the pathology-based diagnosis of lung cancer, cytology is first performed using cells biopsied during a bronchoscopy [4], and comprehensive diagnostic results are then obtained from histological examinations. However, there are considerable variations in cell types, including atypical regenerative tumorous cells. Correspondingly, expert screeners or cytologists sometimes need to make difficult judgments. In addition, the detection of abnormal cells from many cell images is a very difficult task. Therefore, if the identification can be supported using image analyses or artificial intelligence technologies [5–10], diagnostic accuracy could be improved.

We have previously developed a method to classify benign and malignant lung cells using a deep convolutional neural network (DCNN) [11], and have also developed a DCNN-based lung cancer type classification system [12]. The overall accuracies of benign/malignant and lung cancer type classifications were 79% and 71%, respectively. To further enhance this performance, it would be necessary to increase the number of images used to train the CNN. However, in cytology, manual manipulation of a microscope is still one of the most typical techniques used for the assessment of three-dimensional cell morphology, yet digitized imaging is still under development. In cytology, it is necessary to focus on individual cells of interest. Hence, planar scans, such as those used for histological diagnosis, cannot convey the necessary information. Therefore, it is not realistic yet to automatically acquire a large number of images—including depth information—digitally. To improve the discrimination performance, it is necessary to consider a method that can obtain good classification performance with fewer data.

For this purpose, we employ a generative adversarial network (GAN), a deep-learning-based image generation technology comprising a generator and discriminator that work in a competitive manner [13]. The generator tries to generate synthetic images which are misinterpreted by the discriminator as real images, while the discriminator trains to distinguish real images from synthetic images. By repeating these processes, the generator can generate synthetic images that are quite similar to real images. This technology is often applied to medical image processing [14].

Wang et al. proposed metal artifact reduction in CT images by using conditional GAN [15]. Guibas et al. developed a method to output fundus images and segmented blood vessel images using two GANs [16]. Frid-Adar et al. generated small computer tomography (CT) images (64 × 64 pixels) of the liver by using a DCGAN and applied it to the classification, and showed that a CNN using GAN-generated images improved the accuracy of lesion classification by 7% [17]. Han et al. generated 256 × 256 pixel brain MR images by using PGGAN and applied them to automated brain tumor detection, consequently improving the overall classification accuracy by 1% [18]. Previously, our group generated lung nodule patterns in CT images with a GAN and used them to train a DCNN to classify benign and malignant patterns. This showed that this method was effective for the improvement of classification [19,20].

Regarding the application of GANs to pathological images, Song et al. proposed a method to generate histopathological images of bone marrow cells to improve the performance of automated bone marrow cell classification [21]. However, applications to cytology and image generation (in which multiple cells exist) have not been successful. It is difficult to generate images with complex configurations of many objects with a conventional GAN, and it is thus also very difficult to obtain high-resolution images.

Recently, Kerras et al. proposed progressive growing of GANs (PGGAN) to solve the problems encountered with conventional GANs, and succeeded in generating high-resolution images [22]. PGGAN may be able to generate high-resolution images of multiple cells. Therefore, this study aims to generate cytological images using PGGAN, and to improve the classification performance using the generated images.

## Materials and methods

### Outline of proposed method

**Fig 1:**
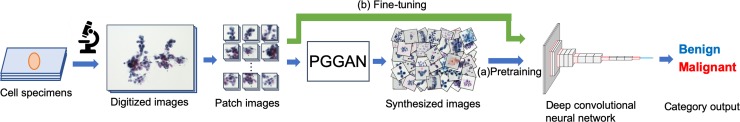
Schematic of the study outline.

The cell classification method proposed in this study is shown in Fig 1. Patch images were segmented from images of cytological specimens acquired with a microscope, and a DCNN classified these images as benign or malignant. DCNN training was conducted in two steps: First, many cytological images were generated by PGGAN, and the DCNN was pretrained on them as shown in Fig 1(A). Subsequently, the DCNN was fine-tuned using actual images, as shown in Fig 1(B).

### Materials

Lung cells of 60 patients were collected with interventional cytology using either bronchoscopy or CT-guided fine-needle aspiration cytology, and comprised 25 benign and 35 malignant cases according to a combined histopathological and immunohistochemical diagnosis. Specifically, biopsy tissues were simultaneously collected with cytological specimens, fixed in 10% formalin, dehydrated, and embedded in paraffin. In some cases, the 3 μm tissue sections were subjected to immunohistochemical analysis. The cytological specimens were prepared with liquid-based cytology using the BD SurePath^TM^ liquid-based Pap test (Beckton Dickinson, Franklin Lakes, NJ, USA) and were stained based on Papanicolaou’s method. Using a digital camera (DP20, Olympus Corporation, Tokyo, Japan) which was attached to a microscope (BX53, Olympus Corporation) with a 40× objective lens, 244 microscopic images of benign cells and 267 images of malignant cells were acquired in a JPEG format with a size of 1280 × 960 pixels per image. This study was approved by an institutional review board and informed consents were obtained from patients subject to the condition of data anonymization (No. HM16–155).

### Image preparation

**Fig 2:**
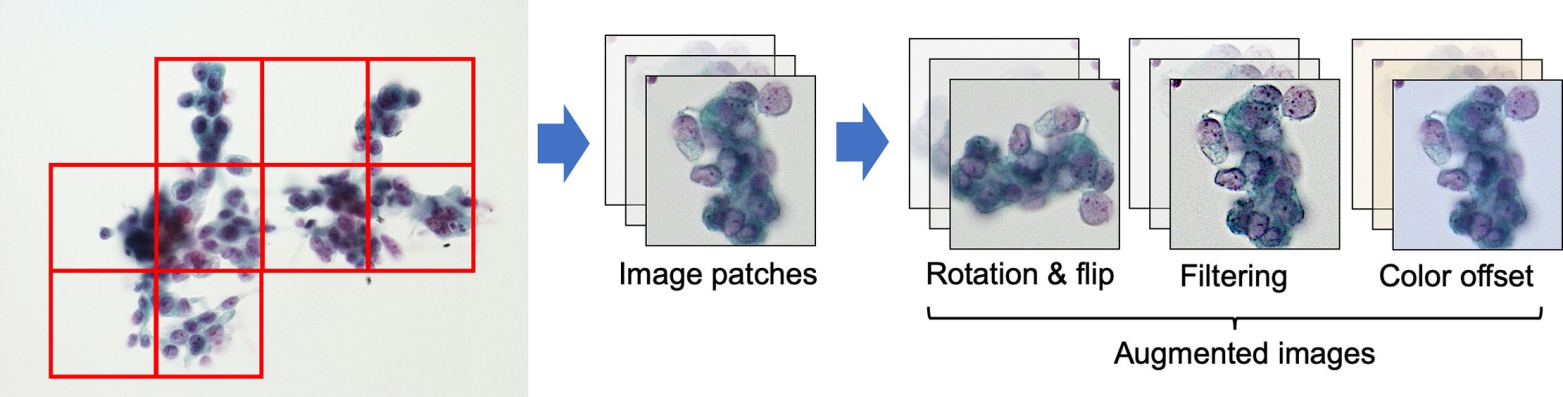
Generation and augmentation of patch images.

The matrix sizes of the acquired microscopic images were too large for direct input to a DCNN. Therefore, the acquired images were divided into nonoverlapping 256 × 256 pixel patches, as shown in Fig 2. The patch images included various cells, which did not necessarily contain malignant cells, even when the images were obtained from a patient with a malignant tumor. Therefore, two cytopathologists evaluated the presence or absence of malignant cells in patch images from all cases of malignant tumors. A total of 793 patch images (391 benign and 402 malignant cells) were obtained. Given that the number of images was still too small to train a GAN and DCNN, rotation (90°, 180°, and 270°), flipping, and color correction, were employed to increase the number of images.

In cytological images, cells often overlap, so they are thicker than the histology. Because we need to focus on each target cell, blurry and sharp places are mixed within the microscopic images. Additionally, because the focus adjustment is performed manually, the image sharpness varies. Therefore, to reproduce the variation in focus, two spatial filters (edge enhancement and a Gaussian filter) were also introduced for data augmentation. Thus, 10,000 benign and 10,000 malignant cell images, including the original images, were generated. This augmentation method was described in detail in one of our previous publication [11].

### Image generation based on PGGAN

A GAN—a new deep learning framework used to estimate generative models via an adversarial process—can be introduced to generate images for training a DCNN. The GAN generator takes a variable extracted from a specific distribution—such as the Gaussian distribution—as input, and synthesizes data based on a multilayer network. In the GAN’s discriminator, either generated or actual data are given to a multilayer network that classifies whether the given data were synthetically generated or real. Deep convolutional generative adversarial networks (DCGANs) are often used when images are generated with a GAN [23]. However, given that the final output image is generated directly from random values, network training becomes unstable, and it is often difficult to generate high-resolution images. Many methods have been developed for the improvement of the quality of GAN-generated images. PGGAN, proposed by Kerras et al., is a method that gradually increases the network layers of the GAN's generator and discriminator and increases their resolutions. In this study, we introduced PGGAN to generate high-resolution images.

**Fig 3:**
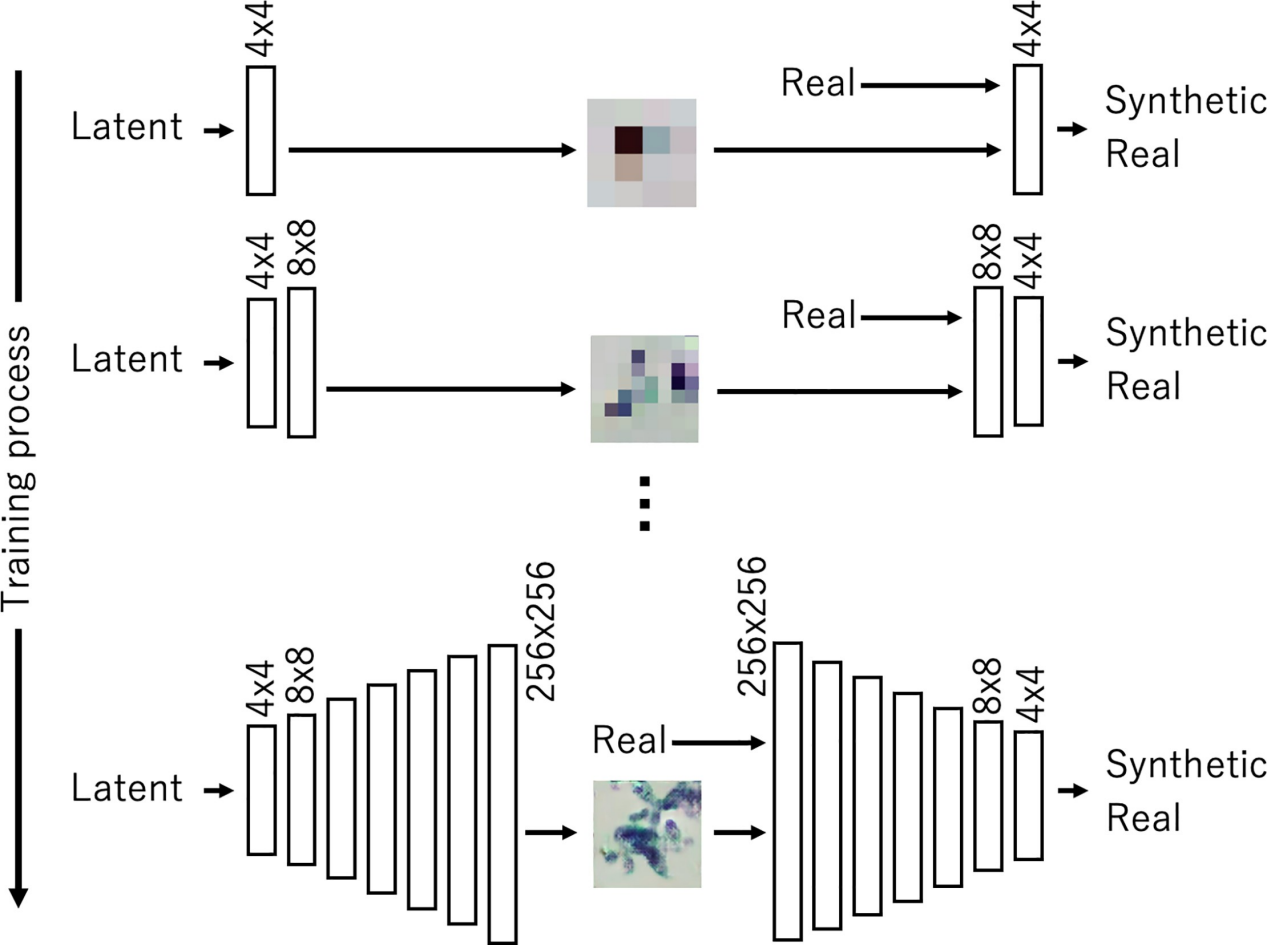
Progressive growing of generative adversarial network (GAN) (PGGAN) training.

As shown in Fig 3, PGGAN initially generates small 4 × 4 pixel images from 128 random values (latent vectors) using a generator with two convolutional layers. The discriminator classifies these images as real or synthetic (i.e., generated). By adding similar networks to the initial networks, PGGAN can then generate and discriminate images that are larger with sizes equal to 8 × 8 pixels. By repeating this addition, high-resolution images will eventually be generated. In this study, PGGAN generated images with sizes equal to 256 × 256 pixels, which are equal to the size as the actual patch images. The PGGAN network structure used in this study was based on the original paper by Kerras et al. [22]. In the convolutional layer, 512 feature maps were used to generate the images with the sizes of 8 × 8 and 16 × 16 pixels. Accordingly, 256, 128, 64, and 32 feature maps were used to generate images with the sizes of 32 × 32, 64 × 64, 128 × 128, and 256 × 256 pixels, respectively. The PGGAN structure was based on WGAN [24], the evaluation functions of the two networks were sliced Wasserstein distances [22]. We adopted the Adam optimizer [25], and set the learning rate to 0.001, β_1_ to 0.0, and β_2_ to 0.999 for 100 training epochs in each scaling step while ensuring that the training progressed stably. We designed two PGGANs to generate benign and malignant cells.

### Two-step DCNN training

**Fig 4:**
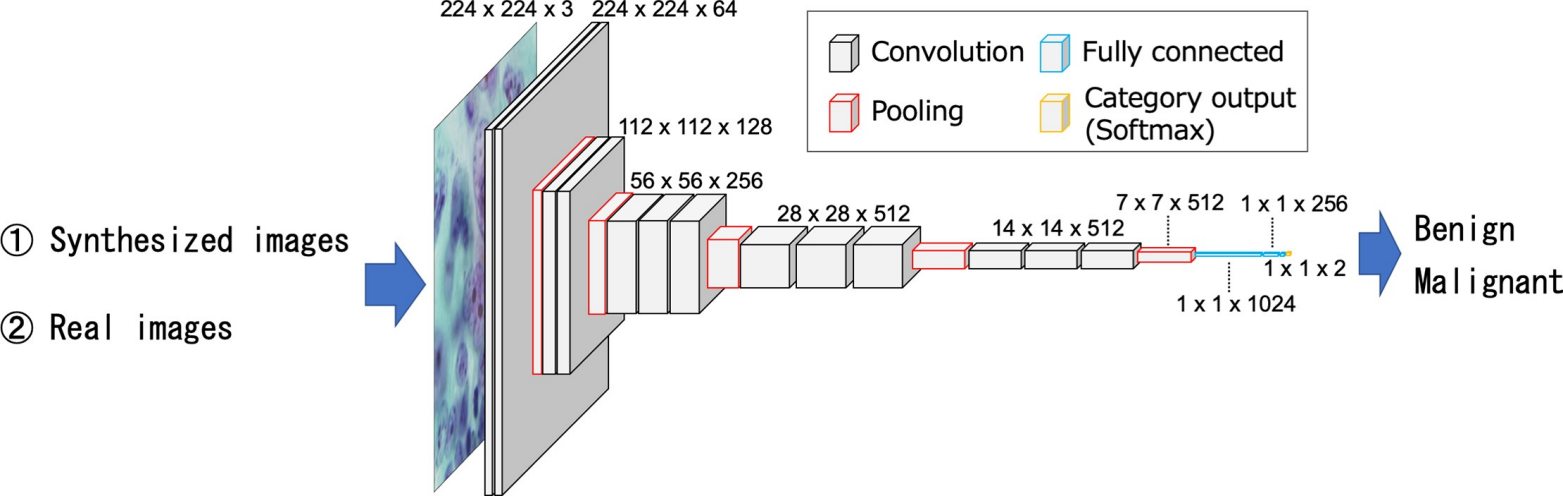
Network architecture used for classification.

A DCNN was used to discriminate between benign and malignant cytological images. In this study, we used the VGG-16 DCNN architecture [26]. VGG-16 is a simple and deep network structure which was released by the Visual Geometry Group at Oxford University in 2014, and ranked second in the 2014 image classification contest [27]. Our previous study confirmed that VGG-16 classified cytological images better than other well-known network architectures, such as AlexNet [5], Inception V3 [28], ResNet-50 [29], and DenseNet [30]. To introduce the VGG-16 network into our study task, we eliminated the fully connected layers from the original network, and new fully connected layers which comprised 1024, 256, and 2 units, were added after the final VGG-16 pooling layer, as shown in Fig 4.

Network training was divided into two steps. First, the image generated by PGGAN was assigned to the DCNN and the weights of the entire network were updated (pretraining). Subsequently, the actual images were input to the trained network, and only the weights of the fully connected layers were updated (fine-tuning).

For these processes, we created an original program using Keras and Tensorflow, we adopted a minibatch size of 32, the Adam optimization algorithm, a learning coefficient equal to 10^−6^, a β_1_ value equal to 0.9, and a β_2_ value equal to 0.999. In the pretraining and fine tuning of DCNN, the same parameters for training were used. The behavior of the training of DCNN for classification was confirmed by using the validation images (173 images), and the common hyperparameters were used thrice.

### Evaluation metrics

This study aimed to a) generate cytological images with a GAN, and b) use them to train a DCNN to improve cytological image classification. To confirm the effectiveness of the proposed method, we analyzed the generated images and the network’s classification performance. First, to confirm the effectiveness of PGGAN, we compared the quality of images generated by PGGAN and by DCGAN, which is a conventional method that has no progressive structure. The DCGAN used in the evaluation consisted of a generator with five convolutional and four scaling layers, and a discriminator with six convolutional and one output layers. The generator generated 256 × 256 pixel images from 128 random values (latent vector). We adopted the Adam optimizer and set the learning rate to 0.00002, β_1_ to 0.5, and β_2_ to 0.999 for 5000 training epochs with a minibatch size of 32 while ensuring that the training progressed stably. Subsequently, we evaluated the DCNN’s classification performance. The final classification performances of the three pretraining methods were compared: a) one was the case in which only images from the ImageNet database were used to pretrain the DCNN, b) the second was the case in which PGGAN-generated images were used, and c) the third was the case in which conventional DCGAN-generated images were used. We created a confusion matrix which compared the network output with the known actual classification. The matrix was calculated by setting a cut-off value of 0.5 for the output of the malignancy of DCNN. Moreover, a cut-off was provided with respect to the DCNN output, classification sensitivity and specificity were calculated, and the receiver operating characteristic (ROC) curves were drawn based on the three conditions to compare the areas under the curves (AUCs).

## Results

### Synthesized cytological images

**Fig 5:**
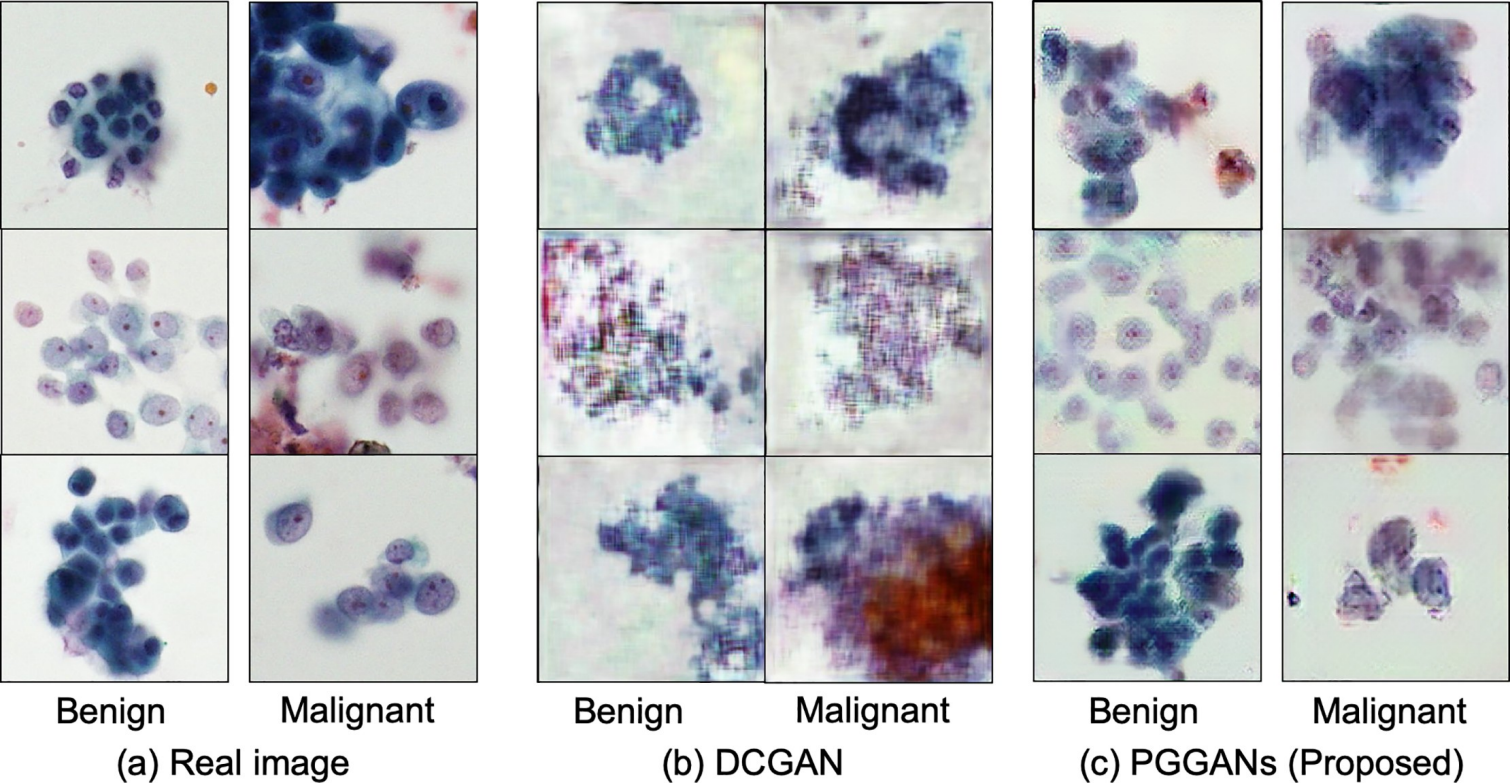
Real and synthesized images.

Fig 5 shows examples of actual cytological images, images generated by a conventional DCGAN, and images generated by PGGAN.

### Classification of benign and malignant cells

**Fig 6:**
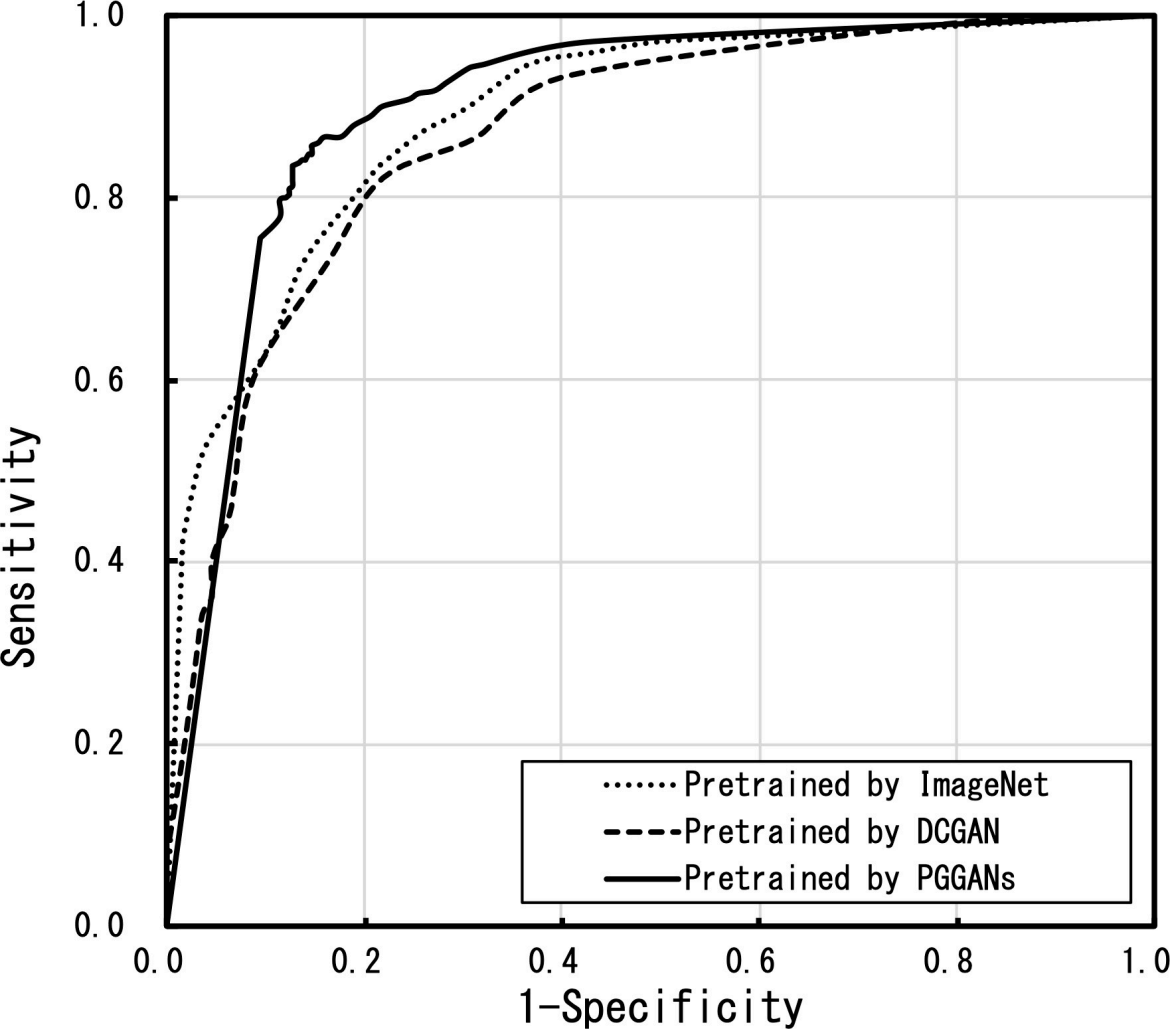
Receiver operating characteristic (ROC) curves of the three pretraining methods.

The pretraining of VGG-16 using the PGGAN-generated images and the classification performance of the DCNN fine-tuned by actual images were evaluated. Table 1 shows the confusion matrix for the classification with threefold cross-validation for a total of 620 images. The total accuracy was 85.3%. Fig 6 shows the ROC curve outcomes. The ROC curves of the DCNN pretrained using PGGAN were better than those using of DCNNs pretrained using ImageNet or with a DCGAN.

**Table 1:** Confusion matrix of the proposed method.

		Estimated		Overall accuracy
		Benign	Malignant	
**Actual**	**Benign**	261	45	0.853
	**Malignant**	46	268	

**Fig 7:**
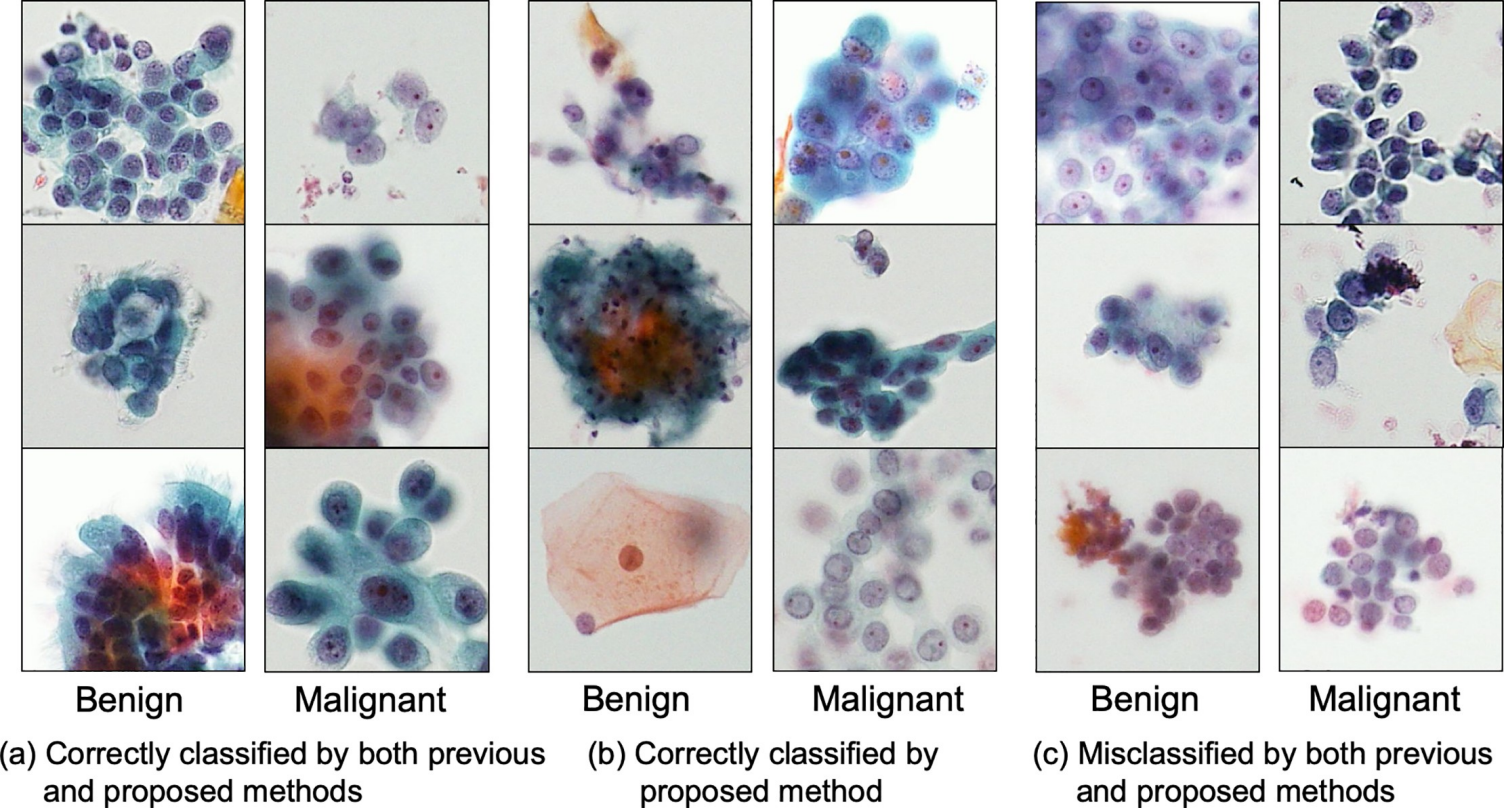
Cells correctly classified and misclassified by the previous and proposed methods.

Table 2 compares the discrimination sensitivity, specificity, overall accuracy, and the AUC of the three methods. The proposed PGGAN method yielded better results than the other two methods. To compare the classified results, Fig 7 shows images correctly identified by both the conventional and proposed methods, images newly identified correctly by the proposed method, and images correctly identified using neither the conventional nor the proposed methods.

**Table 2:** Comparison of pretraining methods.

Pretraining method	Sensitivity	Specificity	Overall accuracy	Az
**ImageNet**	0.850	0.768	0.810	0.872
**DCGAN**	0.793	0.797	0.795	0.867
**PGGAN**	0.854	0.853	0.853	0.901

## Discussion

In this study, we improved lung cell classification using GAN-generated images of benign and malignant cytological images. We introduced PGGAN, a GAN-related technology capable of generating high-resolution images, as well as a two-step learning pretraining process with GAN-generated images, which was fine-tuned with actual images.

In the cytology, the size of the nucleus, the proportion of the nucleus in the cell, the amount of chromatin, and the nucleolus are cleary observed to evaluate the degree of cell atypia. Additionally, the shape of the cell clump and the color of the cell are observed for cell type classification. From these perspectives, the generated images of PGGAN and DCGAN were subjectively evaluated by a pathologist. In the image generated by DCGAN, which is a conventional method, the shapes of the cell clump were mostly reproduced; however, the outline and internal structure of the cells were unclear. Therefore, it is very difficult to obtain the image for the evaluation of malignancy using the conventional DCGAN. On the other hand, the images generated by PGGAN reproduced the shape of the cell clumps and the texture of the nucleus and cytoplasm. However, more detailed cell morphology is required to determine the atypism of cells in the diagnosis. The color reproducibility of the images obtained by both PGGAN and DCGAN was acceptable.

As described above, although the image generated by PGGAN does not have sufficient quality for use in the evaluation of malignancy and cell types, the image quality was better than that of the image generated by DCGAN; the basic structure and sequence were reproduced to withstand for the pretraining of DCNN.

Use of PGGAN-generated images to pretrain a DCNN improved the classification specificity by approximately 8.5% while it retained the detection sensitivity as compared with the use of the conventional method of pretraining with the ImageNet database. The detection sensitivity and the overall classification accuracy of the DCNN, which was pretrained using DCGAN-generated images, were the lowest of the three pretraining methods possibly because the DCGAN-generated images did not contain sufficient shape and color information. The AUC of the proposed PGGAN method was the highest and confirmed the effectiveness of pretraining the DCNN using GAN-generated images. Images synthesized by DCGAN had insufficient quality in the cell nucleus, and the edge and contrast information constituting the cell morphology was poor. Therefore, pre-training using DCGAN generated images is considered to be inferior to pre-training using ImageNet. On the other hand, images synthesized by PGGAN had a good representation of cell nuclei and layout, as well as cell patterns not included in the training image; they are used effectively for pretraining the DCNN.

Deep learning using a DCNN requires many images to evoke a good performance. However, it may be difficult to obtain a sufficient amount of medical image data. Experimental results indicate that our scheme will be useful for the classification of medical images even when only limited data are acquired. In this study, cytological images were classified as benign or malignant. However, many atypical cells have intermediate characteristics. Therefore, in the future, it will be necessary to consider methods that classify cells into three types, including atypical cells.

## Conclusions

In this study, we developed a method to automatically generate cytological images with the use of a GAN and performed a two-step learning process with the use of a DCNN to improve the classification of benign and malignant cytological images with the use of a DCNN. In the proposed method, patch images were segmented from the original microscopic images which allowed the generation of numerous high-resolution images with PGGAN. We also pretrained a VGG-16 DCNN with the use of the synthesized images and fine-tuned the DCNN with actual patch images. In an experiment with 793 patch images, the proposed method improved the classification specificity by 8.5% and the total classification accuracy by approximately 4.3% compared to a network which was fine-tuned with ImageNet. These findings confirmed the effectiveness of the use of PGGAN-generated images.
